# The Knowledge of Traumatic Dental Injury Management Among Dentists in Casablanca-Settat, Morocco: A Cross-Sectional Study Based on the 2020 International Association of Dental Traumatology (IADT) Guidelines

**DOI:** 10.7759/cureus.98059

**Published:** 2025-11-29

**Authors:** Malika Afif, Jirrari Lina, El Arabi Samira

**Affiliations:** 1 Pediatric Dentistry, Faculty of Dental Medicine, University Hassan II, Ibn Rochd Hospital, Casablanca, MAR; 2 Dentistry, Mohammed VI Faculty of Dental Medicine, Mohammed VI University of Health and Medical Sciences, Casablanca, MAR

**Keywords:** dental practitioners, dental trauma, guideline, international association of dental traumatology, knowledge

## Abstract

Background and objective

Traumatic dental injuries in children are a major global public health issue and are among the most common orofacial traumas. They compromise oral health and substantially affect children’s quality of life. This study aimed to assess the knowledge levels of private dental practitioners based on the 2020 guidelines of the International Association of Dental Traumatology (IADT) to identify existing gaps and propose strategies to improve the management of traumatic dental injuries in permanent teeth among children.

Materials and methods

This cross-sectional study was conducted among 354 dentists practicing in private clinics in the Casablanca-Settat region, Morocco. Data were collected using a structured questionnaire distributed either in person at dental practices or online through Google Forms. The questionnaire comprised two sections: the first focused on participants’ sociodemographic and professional characteristics, while the second included 12 items evaluating the proper management of traumatic dental injuries in children based on the 2020 IADT guidelines. Data analysis was conducted using SPSS Statistics software (IBM Corp., Armonk, NY).

Results

Of the 384 questionnaires distributed, 354 dentists responded, yielding a response rate of 92.18%. Of note, 83.6% had already treated one case of dental trauma in children, and 9.4% of them had treated 10 or more cases in the last 12 months. The rate of correct answers was poor, or even average, for the questions on dental expulsion: immediate management (39.8%), the solution for preserving the expelled tooth (62.4%), the optimal time to start endodontic treatment on an immature tooth (56.4%), mature tooth (33.3%), and finally, the duration of retention in the case of dental expulsion without associated alveolar fracture (23.2%). As far as fractures are concerned, more satisfactory rates were observed: the course of action to be taken in the event of a root fracture (67.5%), and in the case of an amelodentin fracture with pulp exposure (66.7%), the duration of follow-up for simple fractures (75.4%), and the factors to be taken into consideration in the event of a corono-radicular fracture (88.4%). Low good response rates were observed for subjects with lateral dislocation with associated alveolar fracture, intrusion, and subluxation (14.4%, 32.8% and 49.4% respectively).

Conclusions

The level of knowledge of dentists in the Casablanca-Settat region in terms of dental trauma in children was found to be acceptable. However, enhancing this knowledge remains important, particularly through encouraging the use of the Dental Trauma Guide (DTG) and promoting continuing education and scientific research.

## Introduction

Traumatic dental injuries are considered a global dental public health problem [1] and are among the most common orofacial injuries [2]. They primarily affect the upper central incisors and most commonly result in crown fractures [3]. Such injuries can significantly impact children’s quality of life, leading to aesthetic, psychological, social, and therapeutic challenges [4]. The management of traumatic dental injuries in children focuses on preventing infection of the dental germ, controlling pain, and reducing sequelae and complications [5]. A study conducted at the Dental Consultation and Emergency Department of the Ibn Rochd Dental Consultation and Treatment Center (CCTD) in Casablanca reported that complications related to dental trauma accounted for 13% of pediatric consultations, ranking third after pain and aesthetic concerns [6].

The International Association of Dental Traumatology (IADT) guidelines are an essential resource for dentists, helping them maintain up-to-date knowledge and provide optimal care for their patients. These guidelines also serve as a fundamental teaching resource for dental students, ensuring they receive solid and regularly updated training in the management of traumatic dental injuries. First released in 2001 and subsequently revised in 2007 and 2012, the most recent version was published in 2020. This update presents four evidence-based treatment guidelines for traumatic dental injuries [7], developed through comprehensive literature reviews and expert consensus discussions [8].

Several studies have assessed the level of knowledge of dentists regarding the management of traumatic dental injuries according to the 2020 IADT guidelines. Findings indicate a moderate level of knowledge among Brazilian practitioners [3], whereas dentists in Australia demonstrated a more satisfactory level [9]. In Morocco, previous research has shown that dentists’ knowledge and practices concerning the management of dental avulsion were also moderate [10]. The present study aimed to assess the level of knowledge among private-sector dentists in Casablanca, Morocco, regarding the management of traumatic dental injuries to permanent teeth based on the 2020 IADT guidelines.

## Materials and methods

This cross-sectional study was conducted among dentists practicing in the private sector in the Casablanca-Settat region who were registered with the National Order of Dentists. A total of 384 dentists were included in the study, based on a sampling calculated with a 95% confidence interval (CI). This study was evaluated and approved by the ethics committee of the Faculty of Dentistry of the Mohammed VI University of Health Sciences. The present research was conducted in accordance with ethical standards, including the 2013 version of the World Medical Association’s Declaration of Helsinki and the additional requirements of Moroccan legislation.

Data were collected through a structured questionnaire, which was distributed either in person at dental offices or online via Google Forms for geographically distant participants. The questionnaire used in this study was adapted from the instrument developed, tested, and validated by Hartman et al., and later translated into English and applied by Jadav et al. [3,9], allowing for direct comparison of results. The first section, addressing sociodemographic characteristics, was modified to reflect the Moroccan context. The second section retained the original questions but was adapted to align with the 2020 IADT guidelines, translated into French, and pretested with dentists to ensure clarity and comprehensibility.

This questionnaire comprised two parts; the first concerned the sociodemographic characteristics of the participants (gender, age, origin of the diploma, specialty, experience in the management of dental trauma cases, and self-evaluation of their knowledge). The second part included 12 questions regarding the management of the traumatized child according to the 2020 guidelines of the IADT. Data were analyzed using SPSS Statistics software (IBM Corp., Armonk, NY) with a significance level of α<0.05.

## Results

Of the 384 questionnaires distributed, 354 dentists responded, representing a response rate of 92.18%. Of note, 58.5% of the participants were female, and 32.8% were between 30 and 40 years old. Regarding their training, 78.2% were graduates of the Faculty of Dentistry of Hassan II University in Casablanca, and 44.4% had obtained their diploma between 2013 and 2022, reflecting one to 10 years of professional experience. In terms of specialties, 44.4% of the practitioners surveyed were specialists, with 49% specializing in dentofacial orthopedics and 8.9% in pediatric dentistry. Most participants (83.6%) had previously managed at least one case of dental trauma, while 9.3% had handled 10 or more cases. When evaluating their own knowledge of dental trauma, 45.2% considered it acceptable, and only 3.7% rated it as perfect (Table 1).

**Table 1:** Sociodemographic and professional characteristics of the participants FMDC: Faculté de Médecine Dentaire de Casablanca; FMDR: Faculté de Médecine Dentaire de Rabat; UIR: Université Internationale de Rabat; UM6SS: Université Mohammed VI des Sciences de la Santé

Variables	N (%)
Sex	
Male	147 (41.5)
Female	207 (58.5)
Age, years	
<30	86 (24.3)
Between 30 and 40	116 (32.8)
Between 40 and 50	87 (24.6)
Between 50 and 60	52 (14.7)
Over 60	13 (3.7)
The origin of the initial training diploma	
FMDC	277 (78.2)
FMDR	19 (5.4)
UIR	3 (0.8)
UM6SS	4 (1.1)
Other	51 (14.4)
The period in which the initial training diploma was obtained	
2013-2022	157 (44.4)
2003-2012	80 (22.6)
1993-2002	81 (22.9)
1983-1992	36 (10.2)
Qualification	
General practitioner	197 (55.6)
Specialist	157 (44.4)
Specialty	
Paediatric dentistry	14 (8.9)
Conservative dentistry and endodontics	6 (3.8)
Oral surgery	32 (20.4)
Periodontist	17 (10.8)
Dentofacial orthopedics ODF	77 (49)
Prosthodontics	11 (7)
The origin of the specialty diploma	
Morocco	76 (48.4)
Obtained abroad	81 (51.6)
The period the specialty diploma was obtained	
2013-2022	75 (47.8)
2003-2012	44 (28)
1993-2002	26 (16.6)
1983-1992	12 (7.6)
Practitioners' experience in treating dental trauma in children	
Never treated a case of dental trauma in children	58 (16.4)
Already treated a case of dental trauma in children	296 (83.6)
The number of cases of pediatric dental trauma treated in the last 12 months	
None	50 (16.9)
1 case	53 (17.9)
2 to 4 cases	130 (44)
5 to 9 cases	35 (11.8)
10 or more cases	28 (9.3)
Self-assessment of knowledge on dental trauma	
Poor	29 (8.2)
Acceptable	160 (45.2)
Good	152 (42.9)
Perfect	13 (3.7)

Each question and its corresponding correct answer were developed based on the latest IADT guidelines concerning the management of traumatic dental injuries.

**Figure 1:**
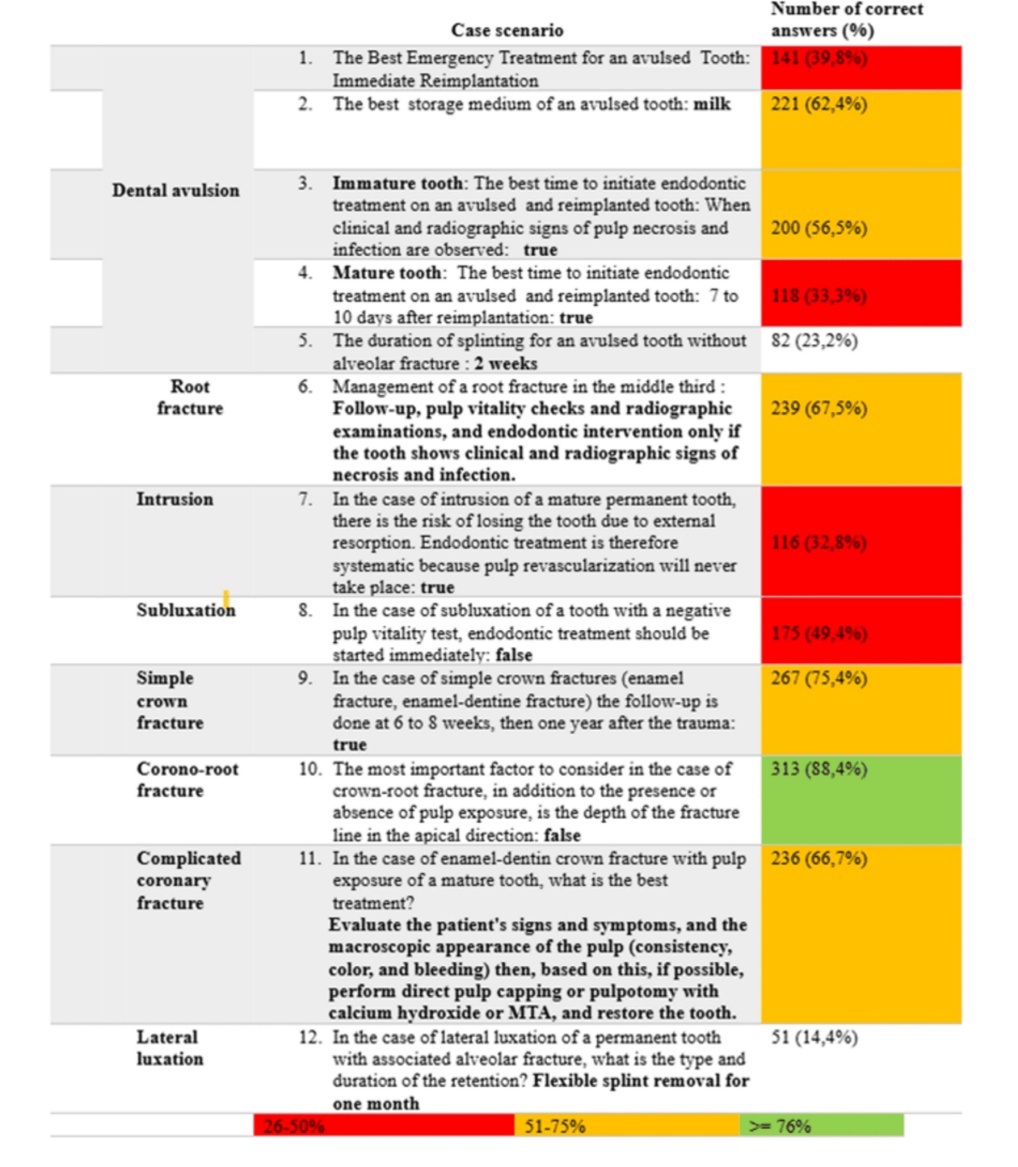
Number of correct answers among the 354 participants for the 12 questions on traumatic dental injury management according to IADT guidelines using a color-coded traffic light system IADT: International Association of Dental Traumatology; MTA: mineral trioxide aggregate

Figure 1 shows the number of correct answers per question, with color coding applied using the traffic light system. Questions 9 (coronoroot fracture) and 10 (simple crown fractures) had the highest numbers of correct answers (88.4% and 75.4%, respectively). In comparison, question 5 (expulsion without associated alveolar fracture) and question 12 (lateral luxation) had the lowest numbers of correct answers (23.2% and 14.4% respectively).

## Discussion

This cross-sectional survey aimed to assess the knowledge of dentists in the Casablanca-Settat region regarding dental trauma in children, based on the latest IADT guidelines (2020), and to examine the different management approaches used in treating traumatized teeth. Our sample showed a predominantly female participation (58.5%), consistent with findings from Azzahim et al. in Rabat, Morocco, where 56.1% of respondents were women. This pattern is not exclusive to Morocco; similar trends have been reported in international studies conducted in the United Arab Emirates, Spain, and Malaysia, where female participation rates reached 68%, 69.8%, and 75%, respectively. A Brazilian study suggested that the growing feminization of the dental profession may be attributed to women's preference for interpersonal interactions and the flexibility offered by the profession [10,11,12,13].

The present study showed that 55.6% of respondents were general practitioners, while 44.4% were specialists. It is important to note that some participants identified themselves as specialists without having completed a residency program, which may have led to an overestimation of this group. In Rabat, Morocco, Azzahim et al. showed that 26.3% were specialists [10], compared to 17.2% in the study by Jadav and Abbott in Australia [9], and 22.3% in the study by Hartmann et al. in Brazil [3]. Regarding the types of specialties, 48.7% of the specialists in our sample practiced dentofacial orthopedics (ODF), and 8.9% were pediatric dentists. These results are similar to those from Rabat, where 61.1% of specialists were in ODF and 11.1% in pediatric dentistry [10]. The high percentage of dentists (83.6%) who had already managed cases of traumatic dental injury in children is particularly advantageous for our study. Conversely, the 16.4% who had never treated such cases may reflect their area of specialization or the specific characteristics of their patient population.

Analysis of the responses of the 296 dentists who had previously treated a case of traumatic dental injuries in children revealed a notable diversity in clinical experience. Overall, 16.9% of practitioners reported no cases in the past 12 months, 17.9% reported one case, and 44% reported between two and four cases. In addition, 9.4% of participants had indicated considerable experience treating more than 10 cases per year. In the Jadav and Abbott study conducted in Australia, 21.1% of respondents had treated 10 or more cases of dental trauma, in adults and children combined, in the past 12 months [9]. These practitioners had significantly higher knowledge scores than those who had not treated any cases of trauma. Similarly, Hartmann et al. in Brazil found that 35.9% had treated more than five cases in the preceding 12 months and noted a proportional relationship between the number of dental trauma cases previously managed and dentists’ competence in this area. This correlation suggests that increased clinical exposure may contribute to improved skills in managing dental trauma [3].

Regarding the self-assessment of knowledge about dental trauma, nearly half (160 practitioners, 45.2%) considered themselves to have acceptable knowledge, reflecting a satisfactory level of competence. In addition, 152 practitioners (42.9%) reported having good knowledge, highlighting solid expertise on the subject. However, only 8.2% of practitioners felt they had poor knowledge, while 3.7% felt they had perfect knowledge. This latter proportion is lower than that reported in Turkey (7.8%), Australia (11.7%), and Brazil (9%) [14,9,3]. A 2017 study by Dhaimy et al. in Casablanca found that 17.7% of participants considered their knowledge insufficient, 68.5% deemed it sufficient, and 13.8% believed it was complete [15]. These findings underscore the importance of implementing continuing education programs on the management of dental trauma. Regarding the urgent management of an avulsed tooth, the present study showed that 39.8% of the participants had given the correct answer, namely, immediate reimplantation at the place of the accident. In response to the same question, 59% of Brazilian and 83.9% of Australian participants answered correctly, indicating a higher level of knowledge [3,9].

As for the best storage medium, 62.4% of participants correctly identified milk as the best storage medium for an avulsed tooth, a result that is more favorable than the 46.7% reported in a similar study conducted in Australia [9]. In the study by Azzahim et al. in Rabat, Morocco, 63.9% of respondents selected milk or saline solution [10]. When compared with international data, the correct response rate remains noteworthy, though slightly lower than figures reported elsewhere: 74.7% in Italy in the 2020 study by Mazur et al. [16], 88% in Brazil according to Hartmann et al. [3], 79.7% in Malaysia in the study by Abdullah et al. [13], and 76.7% in Turkey in the 2019 study by Sevencan et al. [14]. According to the 2020 IADT guidelines, the most suitable and practical storage media, in descending order, are milk, physiological saline, saliva, and saline solution. Although water is a less favorable medium due to its low osmolarity, it is still preferable to let the tooth dry in the open air [17]. The IADT has developed the "Save Your Tooth" poster as an educational tool offering guidance on how to manage trauma to a permanent tooth in children, including fractures and avulsions [17].

Of note, 56.6% of participants in the present study correctly answered the question regarding the optimal time to initiate endodontic treatment of an immature tooth that has been avulsed and reimplanted, stating that treatment should begin once clinical and radiographic signs of pulp necrosis or infection appear. In comparison, 70% of respondents in Australia and 51% in Brazil answered this question correctly [3,9]. The goal of reimplanting immature teeth in children is to achieve pulp revascularization and complete apexogenesis. The risk of root resorption due to external (inflammatory) infection should be weighed against the chances of revascularisation, as this resorption is particularly rapid in children. In the absence of spontaneous revascularization, options such as apexification, pulp revitalization/revascularization, or root canal treatment should be initiated as soon as pulp necrosis and infection are diagnosed [17].

As for the optimal time to initiate endodontic treatment on a mature tooth, the results of our study indicated a notable divergence of opinion among the participants. Although a majority (33.3%) gave an incorrect answer, nearly half of the participants (48.3%) considered the statement correct, believing that treatment should begin 7-10 days after reimplantation. These findings are consistent with results reported in Australia [9]. This response pattern can be attributed to recommendations in earlier versions of the IADT guidelines. Previous studies conducted in Brazil (2016), Malaysia (2016), Turkey (2018), Spain (2019), and Rabat (2019), which were also based on earlier IADT guidelines, reported that 48%, 39.9%, 61.2%, 51.7%, and 52.7% of participants, respectively, recommended initiating endodontic treatment 7-10 days after reimplantation, which at the time was considered correct [3,10,12,13,14]. These findings closely align with the results observed in our study.

The latest version of the IADT guidelines states that treatment should be initiated within two weeks of reimplantation. The protocol involves placing calcium hydroxide in the root canal for one month, followed by a permanent root canal filling. If a corticosteroid or corticosteroid/antibiotic mixture is chosen as an anti-inflammatory and anti-resorptive intracanal medication, it should be placed immediately or shortly after reimplantation and left in place for at least six weeks. Medications should be applied carefully into the root canal, avoiding placing them in the crown of the tooth. Some medications can cause discoloration of the teeth, leading to patient dissatisfaction [17].

Regarding splinting duration, 23.2% of participants selected the appropriate two-week period, while 68.6% opted for a six-week duration, which increases the risk of ankylosis and replacement resorption [9]. The results of our study follow a similar trend to that observed in the study by Azzahim et al., where only 22% of participants opted for a restraint duration of 7-14 days [10]. Comparing our results with studies conducted in other countries, significant disparities emerge. In Spain and Turkey, 42% and 65.9% respectively, opted for a splinting duration of 7-14 days [12,14], while in the United Arab Emirates and Malaysia, the majority (69.9% and 64.8% respectively) opted for a splinting removal of 7-10 days [11,13]. In Australia, 57.8% of respondents selected a two-week duration, aligning with current guideline recommendations [9].

In the case of a root fracture in the middle third, follow-up, pulp vitality checks, and endodontic intervention should be performed only if the tooth shows clinical and radiological signs of necrosis and infection. Of note, 67.5% of the participants answered this question correctly. These results are comparable to those reported by Jadav and Abbott in Australia (85.5%) and Hartmann et al. in Brazil (62%) [3,9]. According to the IADT guidelines, if the crown fragment is displaced, it should be repositioned and stabilized using a passive, flexible splint for four months. It is also advisable to monitor the healing of the fracture for at least one year while monitoring the pulp condition [8]. Pulp necrosis and infection may develop later, mainly in the coronal fragment, which may necessitate endodontic treatment specific to this segment. The apical segment rarely experiences infectious complications [8].

When asked about the management of intrusion in a mature permanent tooth, 32.8% of the participants said that there is a risk of losing the tooth due to external resorption, and that endodontic treatment is therefore systematic because pulp revascularization will never take place. Similar results were reported in Australia by Jadav and Abbott (38.9%) and in Brazil by Hartmann et al. (28%) [3,9]. This consistency may be due to the structure of the question, which included four statements to be assessed simultaneously: the risk of tooth loss, external resorption, the routine nature of endodontic treatment, and the chances of pulp revascularization. Such complexity may have contributed to incorrect responses, as participants needed to assess all statements correctly rather than individually. Another possible explanation is a limited understanding, shared by practitioners in different countries, regarding the proper management of intruded teeth, which is concerning. Dental intrusion requires endodontic treatment as an emergency measure, given that this trauma results in severe compression of the neurovascular bundle, leading to pulp necrosis of mature teeth [8,9,18]. Also, it causes damage to the root surface and/or periodontal ligament, leading to external inflammatory resorption [18].

In the case of subluxation of a tooth with a negative pulp vitality test, 49.4% of participants would refrain from initiating endodontic treatment solely based on a negative pulp vitality test response. More favorable results were observed in Australia and Brazil (85.6% and 70% respectively). This aligns with current guidelines, which note that false-negative responses can persist for several months [8]. In the present study, 75.4% of participants correctly opted for follow-up at six and eight weeks, and then at one year, for cases of simple crown fractures. These results are higher than those reported by Jadav and Abbott in Australia (62.8%) and Hartmann et al. in Brazil (67%) [3,9]. The immediate management of simple crown fractures, such as enamel fracture or enamel-dentin fracture, ideally consists of reattaching the fractured tooth fragment. However, depending on the extent, location, and exposure of the dentin, treatment may require ameloplasty, composite reconstruction, or indirect pulp capping with calcium hydroxide or glass ionomer cement, followed by tooth reconstitution [8].

In the case of an enamel-dentin fracture with pulp exposure of a mature tooth, 66.7% of participants would favor an evaluation of the patient's signs and symptoms, as well as macroscopic observation of the pulp, including its consistency, color, and any possible bleeding. These elements then guide the decision to perform, if possible, a direct pulp capping or a pulpotomy, followed by the restoration of the tooth. Comparable findings were reported by Hartmann et al. in Brazil and Jadav and Abbott in Australia, where 79% and 78.9% of participants, respectively, responded correctly, indicating a degree of consistency in clinical management across settings [3,9]. The high rate of correct responses in managing crown fractures likely reflects the frequent occurrence of this type of trauma, which contributes to greater familiarity and knowledge among dentists.

Furthermore, while previous versions of the IADT recommendations indicated that, in patients with mature permanent dentition, endodontic treatment is generally preferred, although pulp capping or partial pulpotomy may also be considered [19], the new recommendations emphasize that conservative treatment is the best option [8]. In the case of lateral luxation of a permanent tooth with associated alveolar fracture, 14.4% of participants correctly indicated flexible splint removal for 30 days, and 40.7% opted for rigid splint removal for 30 days. The study conducted by Hartmann et al. in Brazil showed similar results, where only 17% of participants answered this question correctly [3]. In Australia, Jadav and Abbott found that 35.6% of respondents recommended removal of a rigid splint after 30 days, which was considered correct; the authors suggested that this response might reflect ambiguity in the guidelines [9].

Lateral luxations are among the most common periodontal lesions in the event of dental trauma. The correct diagnosis, followed by the repositioning of the tooth in the correct position, is fundamental for the healing of the periodontal ligament. Incorrect repositioning of the tooth can lead to poor alveolar healing and chronic pain due to apical fenestration [20]. According to the IADT and the American Association of Endodontists, teeth with lateral luxation without associated alveolar fractures should be stabilized using a passive, flexible splint for four weeks, typically with a stainless-steel wire no thicker than 0.4 mm [8,21]. For cases involving alveolar fractures, the IADT also recommends a flexible splint for four weeks. In a multivariate study of 71 teeth with alveolar fractures, neither the type of splint nor the duration of fixation significantly affected the development of pulp necrosis or pulp canal obliteration [22]. Given that teeth with lateral luxation inherently involve alveolar fractures, the splinting recommendation applies consistently to both types of trauma [22].

This study has certain limitations. Potential biases may have arisen from the translation of the questions, their interpretation, and the selection of responses. In addition, methodological differences between studies in other countries do not allow for a standardized interpretation of results, and comparison may prove difficult.

## Conclusions

The results of this study highlight an acceptable level of knowledge among dentists regarding the management of dental trauma based on IADT guidelines. However, some shortcomings remain, particularly in the management of dental avulsion, the duration and type of splint, and the optimal timing of endodontic treatment according to the type and severity of traumatic dental injuries. In order to address these shortcomings, regular participation in continuing education programs focused on dental trauma is essential. Practical workshops and interactive seminars, offering updates on the latest diagnostic and treatment techniques, will significantly contribute to enhancing skills and ensuring that dentists’ knowledge remains current.
